# Weather Variability and COVID-19 Transmission: A Review of Recent Research

**DOI:** 10.3390/ijerph18020396

**Published:** 2021-01-06

**Authors:** Hannah McClymont, Wenbiao Hu

**Affiliations:** School of Public Health and Social Work, Institute of Health and Biomedical Innovation, Queensland University of Technology, Brisbane, QLD 4059, Australia; w2.hu@qut.edu.au

**Keywords:** COVID-19, weather, temperature, humidity, precipitation, wind speed, seasonality

## Abstract

Weather and climate play a significant role in infectious disease transmission, through changes to transmission dynamics, host susceptibility and virus survival in the environment. Exploring the association of weather variables and COVID-19 transmission is vital in understanding the potential for seasonality and future outbreaks and developing early warning systems. Previous research examined the effects of weather on COVID-19, but the findings appeared inconsistent. This review aims to summarize the currently available literature on the association between weather and COVID-19 incidence and provide possible suggestions for developing weather-based early warning system for COVID-19 transmission. Studies eligible for inclusion used ecological methods to evaluate associations between weather (i.e., temperature, humidity, wind speed and rainfall) and COVID-19 transmission. The review showed that temperature was reported as significant in the greatest number of studies, with COVID-19 incidence increasing as temperature decreased and the highest incidence reported in the temperature range of 0–17 °C. Humidity was also significantly associated with COVID-19 incidence, though the reported results were mixed, with studies reporting positive and negative correlation. A significant interaction between humidity and temperature was also reported. Wind speed and rainfall results were not consistent across studies. Weather variables including temperature and humidity can contribute to increased transmission of COVID-19, particularly in winter conditions through increased host susceptibility and viability of the virus. While there is less indication of an association with wind speed and rainfall, these may contribute to behavioral changes that decrease exposure and risk of infection. Understanding the implications of associations with weather variables and seasonal variations for monitoring and control of future outbreaks is essential for early warning systems.

## 1. Introduction

**Figure 1:**
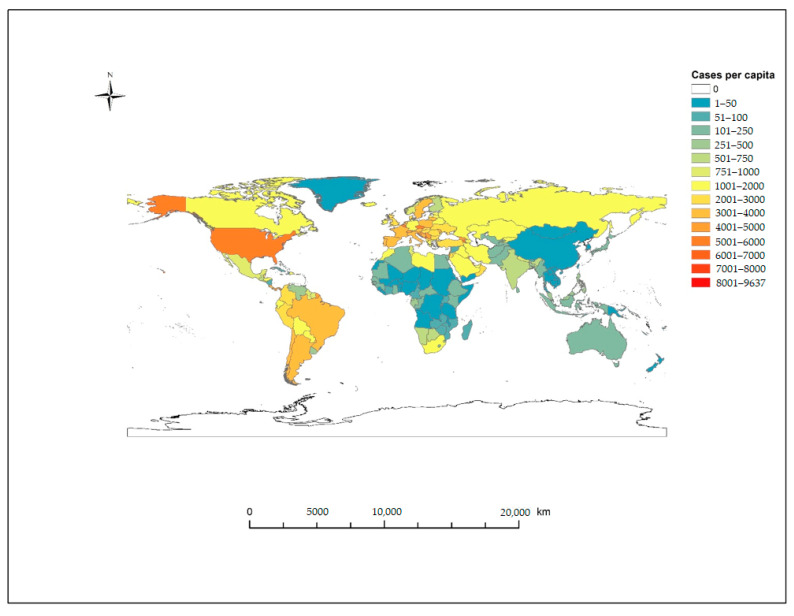
Global distribution of COVID-19 cases per capita as of 16 December 2020 (cases per 100,000 population).

In December 2019, the World Health Organization (WHO) was alerted to cases of atypical pneumonia with unknown etiology in the city of Wuhan, Hubei Province, China. The disease, termed COVID-19 (Coronavirus Disease 2019) spread by human-to-human transmission from China throughout Asia and into Europe, North America, South America and Oceania and declared a pandemic by the WHO on 11 March 2020 [1,2]. As of 16 December 2020, over 74.7 million cases have been confirmed in 214 countries and territories, with over 1.65 million deaths recorded as a result of COVID-19 [3].The three most affected countries account for 45.7% of all cases globally and include the US, with 23% of all cases (*n* = 17,163,944), India with 13.3% of cases (*n* = 9,956,557) and Brazil with 9.4% of all cases (*n* = 7,040,608) and 38.5% of total global deaths from the US (18.7% *n* = 310,095), Brazil (11.1% *n* = 183,735) and India (8.7% *n* = 144,451) (Figure 1).

COVID-19 is a viral respiratory illness caused by the beta-coronavirus SARS-CoV-2, that spreads rapidly through aerosolized droplets and virus-contaminated hands and surfaces [4]. SARS-CoV-2 is from the same family of human beta-coronavirus, identified as causative agents in Severe Acute Respiratory Syndrome (SARS) and Middle East Respiratory Syndrome (MERS), SARS-CoV and MERS-CoV, respectively. The primary mechanism of action of SARS-CoV-2 is through binding with angiotensin-converting enzyme 2 (ACE2) receptors on surfaces of biological membranes predominantly found in the cells of the heart, lungs, arteries, intestine and renal tissues [5]. Following infection with SARS-CoV-2, incubation time can vary from 2–14 days before symptom presentation and predominantly affects the lower respiratory system, with a clinical presentation of dry cough, fever and fatigue [6,7]. The severity of symptoms varies by the individual, ranging from asymptomatic presentation to severe and life-threatening symptoms, including myocardial dysfunction and acute respiratory failure [8,9]. Those most at risk of severe COVID-19 presentation are older individuals and those with pre-existing conditions and multi-morbidities, particularly cardiovascular disease or diabetes [10].

The effects of weather variability on COVID-19 transmission is an emerging area of interest; as COVID-19 has similar transmission modes to other respiratory viruses such as seasonal influenza, it is predicted that SARS-COV-2 could have a similar relationship with weather variables such as temperature, humidity, rainfall and wind speed [11]. Weather and infectious diseases are linked, with the potential for weather variability to favor the emergence of novel viruses and contribute to disease transmission, morbidity and mortality, understanding global spatial and temporal patterns of COVID-19 transmission is vital in the control and prevention of future outbreaks [12]. Due to the widespread and continuing transmission of COVID-19, it is predicted that COVID-19 outbreaks will persist into the future and potentially exhibit a seasonal outbreak profile similar to influenza and other infectious respiratory diseases [13,14].

Understanding the potential for seasonality and the association with weather is particularly relevant in the context of the last pandemic; 1918–1919 Influenza Pandemic, also known as the “Spanish Flu”, caused by an Influenza A virus of avian origin [15]. This outbreak is suspected to have first emerged in the autumn/winter period of 1917 and spread through troop movements and deployments during the First World War across Europe, North America and Asia [16]. This pandemic exhibited three distinct waves of outbreaks with very high mortality rates and virulence associated with cold temperatures and increased precipitation in each of the peaks in Spring 1918, Autumn 1918 and Winter 1918–1919 [17]. Since the 1918 Influenza Pandemic, influenza A and B strains continue to circulate the globe with distinct seasonal patterns of outbreaks associated with varying climate regions, and in recent history, novel viruses have emerged, also showing an association with weather variables—2009 H1N1 (Swine Flu) Pandemic, SARS 2003 and MERS 2012, among these.

Seasonal influenza outbreaks have a distinct seasonal profile, with annual peaks in outbreaks coinciding with winter and the associated cold and dry weather patterns [18]. Seasonal outbreaks in subtropical and tropical climates exhibit a different pattern, often with a persistent low level of cases in the community, with multiple outbreaks over the course of a year, most commonly in the shoulder seasons from Autumn through to Spring [19,20]. These seasonal outbreaks vary in severity and climate and weather has been associated with these variations, with changes in weather favoring increased transmission or contributing to increased morbidity and mortality [21]. Severe and early seasonal outbreaks of Influenza can occur when a cold winter follows a mild winter, with wider variations in weather increasing as a result of a changing climate [22,23]. Another important use for this information is in developing surveillance systems for early detection of COVID-19, to enable the timely and effective implementation of public health measures and lockdowns [24]. As is the case for current influenza surveillance methods, COVID-19 case and cluster detection is limited due to the delay between onset of disease and confirmation through testing. Understanding the association between weather variability and COVID-19 incidence and transmission is vital and can contribute to the development of early warning systems and surveillance for seasonal outbreaks when used in conjunction with “big data” such as internet search queries or Google search trends, for trend forecasting of seasonal weather patterns of transmission and outbreaks [25,26,27].

As a recently emerged disease with a significant global impact on health, there are limited published peer-reviewed studies on the association between COVID-19 and meteorological or climate factors, but the available literature is growing. The purpose of this review is to update and further evaluate available literature on the association between weather variables including temperature, humidity, wind speed or rainfall and COVID-19 incidence over a wide climate range, to provide useful information for predicting seasonality and early warning for future outbreaks.

## 2. Materials and Methods

### 2.1. Search Strategy

This review was conducted according to the Preferred Reporting Items for Systematic Reviews and Meta-Analyses (PRISMA) guidelines. We searched PUBMED, Web of Science and Scopus databases for relevant studies on COVID-19 and weather with the results restricted to journal articles on human studies published in English. The keywords used in this study included “COVID-19” and “temperature” or “humidity” or “wind speed” or “rainfall” or “weather” or “climate” or “seasonality” or “spatial” or “temporal”. Titles and abstracts were scanned for relevance and further relevant studies were identified from references.

### 2.2. Inclusion and Exclusion Criteria

**Figure 2:**
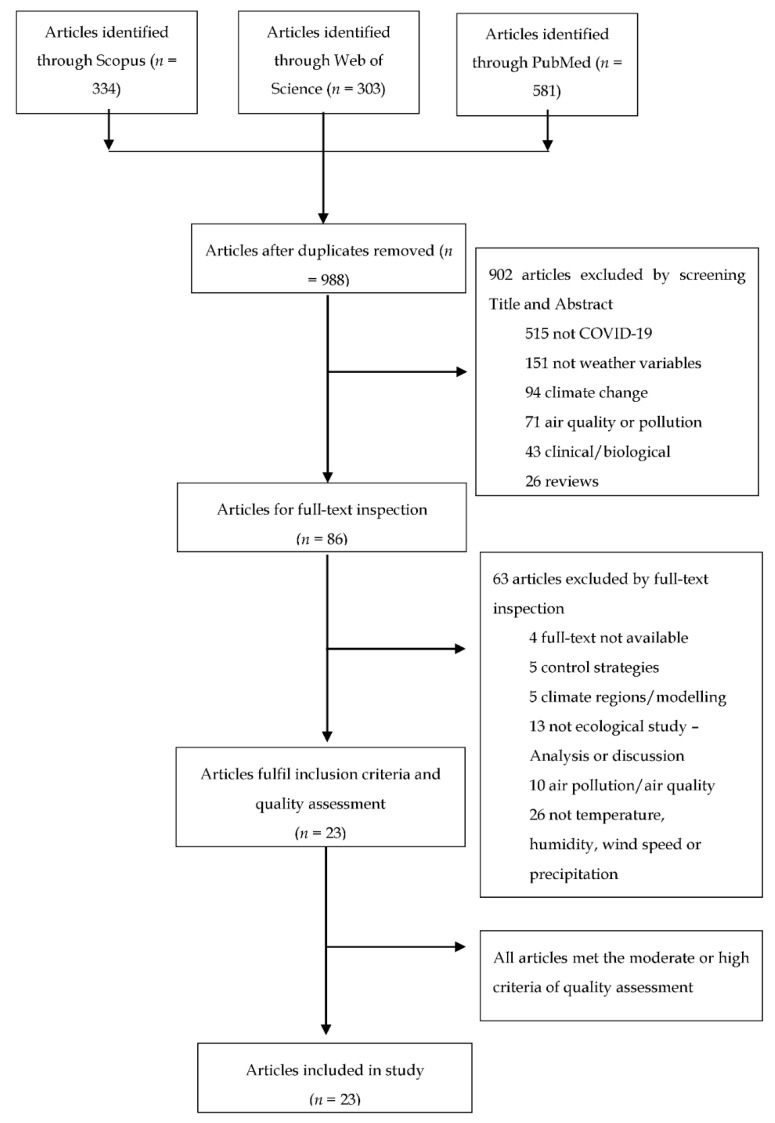
Study selection flow chart.

Eligible articles included epidemiological studies to evaluate the association between weather variables and COVID-19 transmission up to 1 October 2020. Ecological studies, preferably with spatial or temporal methodologies, were considered for inclusion. Letters, experimental studies, reviews and duplicated publications were excluded from potential articles (Figure 2).

### 2.3. Quality Assessment and Data Analysis

Using modified criteria from BioMed Central, the quality of eligible studies was assessed (Supplementary Materials—Table S1) [28]. The studies are ranked according to several areas including: source of information; confounding factors; study design and statistical analysis to evaluate the quality of information. According to the criteria scale, the scoring range is from 1–22 (Low: 1–7; Middle: 8–15; High: 16–22).

All eligible articles were reviewed, and the following information was extracted from these: first author, journal of publication, study site/location, study design, incidence (cumulative cases or daily cases), weather variables (keywords) and source, main findings, strengths, limitations and confounders. Due to the varying study designs, statistical analysis and potential confounders, no meta-analysis was performed.

## 3. Results

### 3.1. Literature Search

From the initial search of PUBMED, Web of Science and Scopus, 1218 articles up to 1 October 2020 were identified by keywords, filtered for English language and studies in humans. After duplicates were removed, 988 articles remained. Studies were then screened by title and abstracts, excluding articles not discussing COVID-19 incidence or weather (i.e., articles focusing on climate change, air pollution or air quality, effect of lockdown on climate or treatment, clinical or biological studies and reviews), further reducing the number of studies to 86 for full-text review. Full-text review reduced the number of articles to 23, after quality assessment evaluating study design, data collection and analysis, discussion and limitations, all 23 articles with moderate or high rating were eligible for discussion. All eligible studies discussed weather variables (temperature, humidity, wind speed, rainfall) and COVID-19 incidence and relationship with community transmission, either spatially or temporally, in a varied range of geographical locations.

### 3.2. Study Characteristics

**Figure 3:**
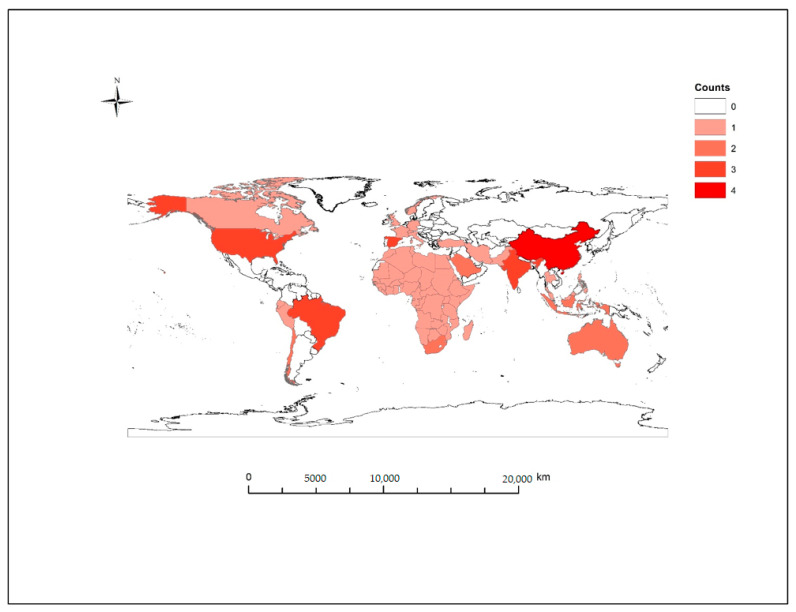
Geographical distribution of locations included in articles for review. (Three global studies of 166 countries, 185 countries and 100 countries were not shown).

All included studies were peer-reviewed and published online by 1 October 2020. Of these studies, four studies were a global analysis of weather variables—two assessing distribution globally [29,30] and one evaluating association in 166 COVID-19 affected countries excluding China [31], and an early analysis of 100 affected countries [32]. The remaining articles were at the continent, country, state or city level (*n* = 19/23) and included three from North America: one from New York, USA [33], one from Canada [34], and the third included selected counties in USA [35]. Four studies from China were included, two at the city level [36,37] and two at the provincial level [38,39], two studies were based in South America—one including multiple countries [40] the second focusing on Brazil [41]. The remaining studies included New South Wales, Australia [42]; a study of countries in Africa [43]; Saudi Arabia [44]; two studies of states in India [45,46]; two studies from Spain [47,48] and three studies at city level including Jakarta, Indonesia [49]; Singapore [50]; and Oslo, Norway [51] (Figure 3). The incidence data included in these studies are categorized from country level to city level, the majority of cases globally were recorded in urban or metropolitan areas—particularly at the beginning of the pandemic, isolated rural and regional areas were less affected in the initial outbreak period and are less likely to be included in the data analyzed. The data sets included in the studies reviewed ranged from 30 days up to 115 days long (mean = 59.3 days, median = 43 days)—the earliest data set started on 1 December 2019–29 February 2020 and the most recent data set ran from 5 March–7 June 2020.

### 3.3. Weather Variables and COVID-19

**Figure 4:**
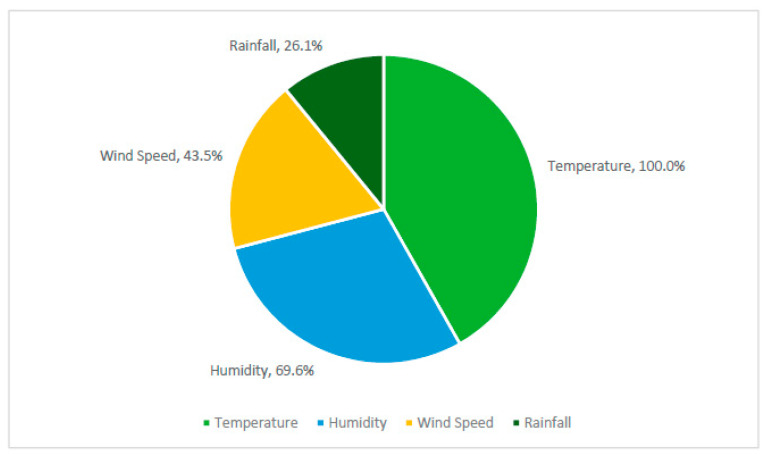
Percentage of studies, including Temperature, Humidity, Wind speed or Rainfall weather variables.

All included articles addressed COVID-19 incidence and weather variability, with all articles including temperature (*n* = 23/23) as the following variables: minimum temperature, maximum temperature, average temperature or diurnal temperature range (Figure 4). Humidity was the next most common weather variable, with 16 of the included studies measuring absolute humidity (AH) or relative humidity (*n* = 16/23). Wind speed was measured in 10 of the included studies (*n* = 10/23), and six studies included precipitation or rainfall (*n* = 6/23).

#### 3.3.1. Temperature

From all 23 articles evaluating the association between temperature and COVID-19, three studies reported no significant association—one study based in Canada, one from Spain and the other from New South Wales (NSW), Australia (Table 1). The authors reported no significant association between temperature and new daily cases [34,42,48]. An optimal temperature range is suggested in Bukhari et al. where the majority of new cases were reported in regions, with the mean temperature recorded between 0–17 °C, while cases for the same period were lower in warm regions (i.e., mean temperature >17 °C). Huang et al. reported 60% of cases occurring in the temperature range 5–15 °C, cases peaked at 11.54 °C. Of the remaining 18 studies, all reported a significant correlation with temperature and COVID-19 incidence; 11 of these were negatively correlated and seven were positively correlated. 

A global study of 166 countries (excluding China) reported a significant negative correlation between temperature and cases, where a 1 °C increase in temperature was associated with a 3.08% (95% CI: 1.53–4.63%) reduction in cases [31]. In the global study including 100 countries in the temperature range −33.9–34.3 °C, the authors reported a significant negative association between daily temperature and global daily cases, when temperatures increased above −15 °C (*r* = −0.88, *p* ≤ 0.001), leading up to 18 March 2020 [32]. In Liu et al., the authors reported a significant negative correlation for both average temperature (AT) and diurnal temperature range in cities in China; the authors reported that the decreases in the daily case counts were 80% (95% confidence interval (CI): 75–85%) and 90% (95%CI: 86–95%), with an increase of 1 °C in daily AT and 1% increase in diurnal temperature range [36]. A time series analysis based on provincial level in China showed a significant association with temperature in Hubei province, where every 1 °C increase in average temperature led to a decrease in daily confirmed cases by 36–57% when accounting for humidity, the association in other provinces varied [38]. Shi et al. reported a significant negative association for provinces in China, with daily COVID-19 incidence lowest at −10 °C and highest at 10 °C [39].

A study in Africa reported that with every 1 °C increase in mean daily temperature, daily cases decreased by 13.53% when accounting for time lag for incubation and infectious periods [43]. In the study from India, for the period 1 April–10 May, the effect of temperature varied across the 11 states, an association with COVID-19 incidence and average temperature was reported as significant in four of the included states—Madhya Pradesh (*r* = 1.43, *p* ≤ 0.05), Maharashtra (*r* = 2.76, *p* ≤ 0.05), Punjab (*r* = 1.49, *p* ≤ 0.05), and Tamil Nadu (*r* = −15.9, *p* ≤ 0.05); maximum temperature was reported as significant in association with COVID-19 incidence in two regions—Maharashtra (*r* = −0.32, *p* < 0.05) and Tamil Nadu (*r* = 0.43, *p* ≤ 0.05), and minimum temperature was reported as significant in association with COVID-19 incidence in two regions—Gujrat (*r* = 0.21, *p* < 0.05) and Uttar Pradesh (*r* = 0.18, *p* < 0.05) [45]. In another study from India, in the three regions of Maharashtra, Rajasthan and Kashmir, Meraj et al. reported significant positive association in two of the three regions analyzed; no significant association between temperature and COVID-19 incidence was reported for Maharashtra (*r* = 0.093); in Rajasthan and Kashmir, a positive association between temperature and COVID-19 was reported (*r* = 0.76, *p* ≤ 0.0001) and (*r* = 0.76, *p* ≤ 0.0001) respectively [46]. In Alkhowailed et al., a significant correlation was reported in Saudi Arabia, for both average temperature (−0.162, *p* < 0.05) and maximum temperature (*r* = −0.211, *p* < 0.01) and daily new cases [44]. In a spatio-temporal analysis of provinces in Spain, Paez et al. found for every percentage point increase in temperature, there was a 1–2% reduction in COVID-19 incidence [47].

In a study of the eight most affected regions and cities in South America, Zhu et al. reported daily average temperature had a strong negative correlation with daily confirmed cases; the strength of this association varied by region, with the city of Santiago reporting the strongest correlation with temperature (*p* < 0.01), while Valparaiso and Lambayeque reported no significant association with temperature [40]. Prata et al. reported a linear negative association between temperature and COVID-19 cases in major cities in Brazil, where every 1 °C rise was associated with −4.9% decrease in COVID-19 cases when the temperature is below 25.8 °C [41]. In a study of New York City, USA, the authors reported a significant association, with minimum temperature (*r* = 0.335, *p* < 0.1) and average temperature (*r* = 0.289, *p* < 0.05) and COVID-19 cases for the period from 1 March–12 April [33]. In a third study based in the US, Chien and Chen reported average temperature as significantly correlated with COVID-19 cases with RR% −0.21 (95%CI: −0.26, −0.15); in further modelling, a threshold of 15.3 °C was identified, where RR% switched from negative to positive above this threshold and peaking at 20.25 °C and decreasing towards 29.2 °C [35]. In an ecological study from Jakarta, Indonesia, Tosepu et al. observed a significant correlation between average temperature and COVID-19 cases (*r* = 0.392; *p* < 0.01), where the average temperature ranged from 26.1 °C–28.6 °C [49]. In Oslo, Norway, both maximum temperature (*r* = 0.347; *p* = 0.005) and average temperature (*r* = 0.293; *p* = 0.019) were significantly positively correlated with COVID-19 cases [51]. Xie and Zhu reported a significant positive correlation with mean temperature in cities in China, where each 1 °C rise was associated with a 4.861% (95%CI: 3.209–6.513) increase in COVID-19 cases when the temperature was below 3 °C [37]. Finally, in Pani et al., for the period from February 4 to May 31, average temperature (*r* = 0.4, *p* < 0.01) and minimum temperature (*r* = 0.32, *p* < 0.01) showed significant positive correlations with new and total COVID-19 cases during this period, while maximum temperature (*r* = 0.40, *p* < 0.01), minimum temperature (*r* = 0.39, *p* < 0.01) and average temperature (*r* = 0.47, *p* = 0) showed strong associations in the early phase of the outbreak from 4 February–30 April [50].

#### 3.3.2. Humidity

Sixteen of the included studies assessed the relationship between humidity and COVID-19, measured as either absolute humidity in g/m³ (18.8% *n* = 3/16) or relative humidity as a percentage (75% *n* = 12/16), or both absolute humidity and relative humidity (12.5% *n* = 2/16). Twelve studies (75%) reported significant associations with relative humidity, absolute humidity or both, with four studies reporting a positive correlation, six studies reporting a negative correlation and two studies reporting an optimal range of humidity for new cases.

The studies from Africa, New York, USA, Jakarta, Indonesia and one global study of 100 countries (*n* = 4/16) did not find a significant relationship between humidity and COVID-19 [32,33,43,49]. Goswami et al. reported mixed results across the regions of India, with positive associations reported in Madhya Pradesh (*r* = 1.211, *p* < 0.05) and Punjab (*r* = 0.584, *p* < 0.05); while a negative association was reported for Tamil Nadu (*r* = −6.79, *p* < 0.05), the authors also reported a significant interaction between temperature and relative humidity [45]. Qi et al. reported a significant negative association with relative humidity and cases in provinces of China, where for every 1% increase in RH, daily cases decreased in the range of 11–22% when AT was in the range of 5.04 °C to 8.2 °C [38]. The authors also reported an interaction between relative humidity and average temperature. In a multivariate analysis from New South Wales, Australia assessing 9 a.m. and 3 p.m. relative humidity, a significant association with humidity was reported, where a 1% increase in 9 a.m. humidity could increase the number of COVID-19 cases by 6.11% [42]. Wu et al. reported an inverse correlation between relative humidity and global daily new cases, where for every 1% increase in humidity, new daily cases reduced by 0.85% (95% CI: 0.51–1.19%) [31]. Alkhowailed et al. reported a weak positive correlation between average relative humidity and new cases in Saudi Arabia (*r* = 0.194, *p* < 0.01) [44]. Chien and Chen also reported a significant positive association with relative humidity in the US (RR 0.07 95%CI: 0.05–0.09) [35]. In Spain, Paez et al. reported a significant negative association between relative humidity and daily cases, with a 3% reduction in incidence per 1% increase in humidity when adjusting for population density, age and transit controls [47].

All studies assessing absolute humidity reported a significant association with COVID-19. Bukhari reported an optimal absolute humidity range, with the majority of reported cases between 1–9 g/m³, and Huang et al. reported 73.8% of confirmed cases in regions with absolute humidity in the range of 3–10 g/m³ [29]. Liu et al. reported a negative correlation between absolute humidity and confirmed case counts across 17 cities in China; when AH increased by 1 g/m³, cases decreased (when adjusted for onset lag of 7 days and 14 days) RR of 0.72 (95% CI: 0.59–0.89) and 0.33 (95% CI: 0.21–0.51) respectively [36]. Zhu et al. reported varying results for absolute humidity, with significant negative correlation for daily confirmed cases reported for Pichincha (*p* < 0.05) and Rio de Janeiro (*p* < 0.01) and significant positive correlation in Santiago (*p* < 0.05) [40]. In Singapore, Pani et al. reported a weak positive correlation with minimum, maximum and average relative humidity (*r* = 0.19, *r* = 0.20 and *r* = 0.21) and COVID-19 cases (*p* < 0.05), with no significant effect during the early phases of the outbreak from February to March, this effect increased in strength with increases in relative humidity (80 ± 4%) in May. Maximum (*r* = 0.27) and average absolute humidity (*r* = 0.59) were reported as having a stronger significant positive correlation with COVID-19 cases compared with relative humidity (*p* < 0.01) [50].

#### 3.3.3. Wind Speed

Wind speed was included or mentioned in ten studies (43.5%). Wu et al., Xie and Zhu and To et al. included wind speed in the model as a confounder and no results were reported [31,34,37]. Of the remaining seven studies, including wind speed as a weather variable, three reported significant associations between wind speed and COVID-19 cases. Adekunle et al. reported a significant positive association with wind speed, where a 1% increase in average wind speed was associated with 11.21% (95% CI: 0.51–1.19) increase in COVID-19 cases in countries in Africa [43]. Pani et al. reported a significant negative correlation with wind speed and COVID-19, where an increase in wind speed is associated with decreased incidence of COVID-19 (*r* = −0.6, *p* < 0.001) [50]. Alkhowailed et al. also reported a significant negative correlation with maximum and average wind speed (*p* < 0.001 and *p* < 0.01, respectively) [44]. Bashir et al., Bukhari et al., Menebo, and Zhu et al. did not report a significant association between wind speed and daily cases of COVID-19 [29,33,40,51].

#### 3.3.4. Precipitation

Precipitation or rainfall was included in six studies (26.1%, *n* = 6/23), no significant correlation was reported for rainfall or precipitation and COVID-19 in studies from New York, USA; Jakarta, Indonesia or NSW, Australia [33,42,49]. To et al. included rainfall as a control variable, no results reported. Chien and Chen reported a significant negative correlation between rainfall and COVID-19 incidence in the US, with daily cases increasing between 1.27–1.74 inches of rainfall and decreasing with rainfall over 1.77 inches of rainfall (<0.0001) [35]. Menebo also reported a significant negative correlation, with daily precipitation levels recorded at 7 a.m. in Oslo, Norway (*p* < 0.05) [51].

## 4. Discussion

The relationship between weather variables and COVID-19 transmission is complex. Exploring association and correlation with weather variables and COVID-19 transmission dynamics is complicated when considering the global scale of a pandemic and additional factors involved in the COVID-19 pandemic including healthcare interventions; public health measures; human behavioral patterns and socio-economic factors. The majority of studies analyzed in this review reported significant associations between weather variables and COVID-19 cases, particularly temperature and humidity, suggesting that weather and climate play a role in transmission dynamics. The overall effect of this association varies, so while seasonal variations and weather patterns may contribute to the increased transmission of COVID-19, other factors such as human behavior and public health measures may play a more significant role in future outbreaks.

### 4.1. Weather Variables and COVID-19

The findings of this review suggest that there is a significant association between both temperature and humidity and COVID-19 incidence, while there is limited evidence for an association between wind speed and precipitation and COVID-19 cases. The significant effect of temperature and humidity on COVID-19 incidence is consistent with findings in earlier studies on airborne respiratory viruses, including SARs, influenza, respiratory syncytial virus (RSV) and MERs [18,52,53].

The studies assessed in this review suggest that ambient or environmental temperature is the most consistently significant weather variable associated with COVID-19 incidence, 90% of the assessed studies reported a strongly significant or significant association with new daily cases of COVID-19, with one study reporting daily cases decreased by 13.53% (95% CI: 1.53–4.63) with a 1 °C increase in mean daily temperature [43]. There was some difference in the range of temperatures between studies reporting negative versus positive correlations with COVID-19 incidence. The differences in temperature ranges could be as a result of short data sets leading to a decreased range of temperature data points over a shorter period of time, particularly during the seasonal transition from winter to spring or summer to autumn. In some regions, low case numbers and limited local transmission were reported at the beginning of the pandemic, particularly in the southern hemisphere and equatorial countries, such as Indonesia, where low case numbers were reported initially, most likely due to low testing capacity, and cases have since increased significantly [54]. Moreover, some research did not control potential confounders (Table 1) and model fit needs to be further improved.

A significant association with humidity was also reported in 66.7% of the included studies, the associations reported varied between positive and negative associations. One study reporting for every 1% increase in relative humidity, new daily cases reduced by 0.85% (95% CI: 0.51–1.19%), while another reported for every 1% decrease in absolute humidity cases decreased by 0.33 (95% CI: 0.21–0.51)—0.72 (95% CI: 0.59–0.89) [31,36].

Temperature and humidity are significant factors in virus transmission and seasonality for several reasons; firstly, these factors determine virus survivability and persistence in the air and on surfaces or fomites [55]. Generally, increased persistence of SARS-CoV-2 and similar viruses is associated with low temperatures and low relative humidity, Chan et al. reported viability of SARS-CoV of over 5 days at temperatures of 22–25 °C and relative humidity of 40–50% on smooth surfaces [56]. A recent study suggested that SARS-CoV-2 may remain viable on glass, stainless steel and banknotes for up to 28 days in optimal temperature conditions of 20 °C, with viability decreasing to 24 h at 40 °C [57]. Outside these optimal ranges, virus survivability is limited, but is sufficient for transmission, as the adaptive immune response is lacking for a previously unknown coronavirus. This leads to a second consideration for the role of weather in transmission, and that is the effect on host susceptibility, where cold dry air inhibits the innate immune response through damage to mucous membranes and slowing of mucociliary clearing [58]. The innate immune response is vital in preventing initial infection, inhibiting viral replication and in mediating the severity of the immune response and inflammation [59].

Following the initial outbreak in Wuhan, COVID-19 spread globally, with the majority of cases recorded in temperate regions in the northern hemisphere experiencing decreased temperatures and humidity [60]. This correlates with an optimal temperature range for transmission proposed by Huang et al. and Bukhari et al., where recorded cases of COVID-19 were significantly associated with temperature in the ranges of 5–15 °C or 0 °C–17 °C and absolute humidity ranges of 1–9 g/m³ and 3–10 g/m³ respectively in the period up to May 2020 or the early stages of spring in the northern hemisphere [29,30]. Since May, as autumn and winter began in the southern hemisphere, cases have increased significantly, particularly in India and South America. The studies included reported significant association with temperature and humidity in these regions [40,41,45,46].

The seasonal patterns of COVID-19 may be similar to influenza, where temperate northern hemisphere regions exhibit a well-defined seasonal outbreak pattern in winter, while tropical and subtropical regions may exhibit a less-defined outbreak over a longer period of time or across multiple seasons—autumn through to spring, as observed in annual influenza patterns [61]. It is vital to understand the effect of weather on COVID-19 transmission for mitigating and preventing future outbreaks, as without significant herd immunity achieved either through a vaccine or exposure or shifting to a less-virulent strain, COVID-19 is likely to continue circulating globally, exerting a significant toll on wellbeing, lives and the economy.

The initial onset or phase one of the outbreak may be delayed in warmer and wetter regions, due to the less optimal conditions for transmission but due to the infectivity of COVID-19 and the lack of existing immunity in the population; these regions will still experience significant outbreaks and mortality rates similar to temperate climates. As the pandemic has progressed, outbreaks have spread to hotter climates and throughout the southern hemisphere—this suggests that hotter countries could experience a lag in the initial outbreak, as hypothesized in a study on climate in Mexico [62], where increased temperature and humidity might slow the rate of transmission initially, but is insufficient to prevent future outbreaks or cause COVID-19 incidence to drop significantly in the summer months.

The observed increase in cases associated with increased temperature, as reported in studies in hotter humid climates (i.e., countries in Africa and South America), may be attributed to many factors separate to weather and climate, such as healthcare infrastructure, hygiene and infection control procedures. Human behavioral patterns may also play a role in transmission, in subtropical or tropical climates with increased humidity and heat people are often gathered indoors in air-conditioned buildings with decreased ventilation and airflow which can contribute to increased transmission risk [63]. This may also contribute to an increase in cases with increased temperature, as described in Menebo, where increased sunshine and warmer weather lead to an increased number of people gathering together in outdoor spaces and subsequent increased risk of exposure and transmission [51]. Air quality and particulate matter (PM) levels are associated with meteorological factors, particularly wind speed. Poor air quality is caused by pollution, urbanization, and exacerbated through climate change with increasing hot, dry weather events and subsequent dust storms and bush fires [64]. Consistent exposure to high levels of PM and poor air quality is a risk factor in respiratory diseases by increasing host susceptibility through oxidative stress and inflammation of the airways contributing to virus transmission and poor respiratory outcomes [65]. High levels of particulate matter also contribute to increased viral survivability and airborne spread of bioaerosols, where large particles deposit on surfaces, while smaller particles remain airborne longer thus increasing risk of transmission; this is particularly relevant with evidence of SARS-CoV-2 RNA detected in particulate matter from outdoor air samples in Italy [66,67].

This research is important for understanding the impact of weather on COVID-19 transmission and can be used to inform and implement health policy for future lockdowns and future vaccine scheduling. Moreover, it may be useful for identifying high-risk behaviors and geographical regions at risk for future outbreaks. This research can also contribute to understanding the changing transmission dynamics as a result of climate change and changing weather patterns and socioenvironmental factors on emerging diseases and future pandemics.

### 4.2. Strengths and Limitations

Due to the growing field of literature available on the COVID-19 pandemic, there are limited reviews available summarizing epidemiological studies of weather and COVID-19. The purpose of this review was to summarize the results and methods of ecological studies on the association between weather variables, specifically temperature, humidity, wind speed and rainfall, and COVID-19 incidence, and provide potential weather predictors for developing an early warning system of COVID-19 transmission. Ecological studies strengths lie in the ability to make comparisons and identify associations at the population level, with the ability to examine spatial and temporal patterns of disease transmission and exposures. The strengths of the included studies are the wide geographical range, including several global studies, as this contributes to the understanding of spatial patterns of transmission globally and association with weather variables in a wide range of climates, looking at the association with COVID-19 outside temperate regions where the pandemic spread initially, particularly in hot and humid climates. Another strength lies in the availability of studies including multiple weather variables, to analyze and better understand possible interactions and associations between weather variables and mediating or confounding weather variables (i.e., interactions between temperature and humidity).

This study has some limitations. While there is an increasing number of studies being published on the relationship between weather and COVID-19, the data available for analysis is limited in several aspects. These limitations include the relatively short period of time since the initial outbreak in January–March for assessing the impact of weather and climate on cases. The sample size ranges of the studies included are based on available data from the beginning of the pandemic and range from 30 days to 115 days, with the earliest data from December 2019 and the most recent in June 2020, therefore it is difficult to assess long term trend and annual seasonality and patterns in climate from a shorter period of analysis. Many countries implemented control strategies to decrease transmission including quarantines, lockdowns and bans on air travel from affected countries by March and April, further influencing the transmission of COVID-19, and may confound or mediate the actual association between cases and weather variables. Ecological studies are prone to ‘ecological fallacy’, where results are interpreted as individual risk rather than group or population risk. Some of the ecological studies included are simple correlation studies, without analysis or modelling to account for confounding, such as lag between test and onset or public health controls.

Confirmed case data may also have limitations due to testing and reporting variations globally, as a result of issues with quality and accuracy of tests in addition to the availability of testing may have created large discrepancies between the actual number of cases and test-confirmed cases, particularly in countries with limited healthcare budgets and infrastructure. The majority of global cases reported from the beginning of the pandemic are also likely to be from urban or metropolitan areas; rural and regional areas are less likely to be represented, particularly in early case counts. When considering the interaction between weather variables and cases, bias may be introduced by using date of case reported and not accounting for the lag or delay between the recorded date of a positive test and initial symptom onset or exposure. Several of the articles included addressed this through statistical modelling with varying lag intervals to account for incubation periods. Another consideration is the reporting of infection sources in terms of locally transmitted cases versus imported cases, where local or community transmission will be influenced by local weather and climate factors.

Finally, the observed association with temperature and humidity may be influenced by the seasonal emergence of COVID-19, where any association with seasonality is coincidental, as the outbreak increased and was transmitted globally through mechanics of population behaviors and epidemic growth rates [68]. The COVID-19 outbreak may have coincided with the northern hemisphere winter and subsided with milder spring and summer weather as a result of public health measures and infection control rather than seasonal forcing [69].

### 4.3. Recommendations for Future Research

Based on this review of the current literature, most of the included studies were ecological, assessing association with temperature, humidity, wind speed or rainfall and COVID-19 incidence. In future research, expanding the models to assess the association of weather with mortality rates and disease severity is an important area for further exploration, also assessing the relationship between temperature and humidity in conjunction with internet search metrics for understanding transmission factors and predicting future outbreaks. Another consideration for future research is the availability of longer data sets (>3 months), as the pandemic continues globally, with the Northern hemisphere reporting record new daily cases moving into the 2020–2021 winter period. Further analysis uses spatiotemporal methods to fully explore the relationship from a spatial and temporal perspective, mapping and modelling, to fully explore the association particularly, as there are now data available over several seasonal transitions. Explore climate zones within countries and regions for spatial patterns.

## 5. Conclusions

The studies included in this review suggest that weather is a significant contributing factor to COVID-19 transmission, particularly temperature and humidity. The relationship between temperature and humidity was addressed in more studies than wind speed and rainfall. Optimal temperature zones were proposed, where the majority of COVID-19 cases were recorded, indicating favorable transmission factors. Wind speed and rainfall may contribute to transmission, but the evidence was not consistent across studies, likely due to limitations in available data and studies addressing these factors. The potential for seasonality and a second wave occurring as a result of the northern hemisphere winter is still emerging. Further analysis of the COVID-19 pandemic moving forward is essential in understanding the impact of weather on transmission and developing early warning systems for future outbreaks, and how this can inform infection control methods and public health measures.

## Figures and Tables

**Table 1:** Characteristics of epidemiological studies on weather variables and COVID-19 transmission.

Record	Journal	Site	Study Period	Source of Case Data	Weather Variables	Study Design	Main Findings	Strengths	Limitations and Confounders
Adekunle et al. 2020 [43]	Heliyon	Africa (urban–rural)	30 March 2020–29 April 2020 (31 days)	Daily confirmed cases from WHO daily situation reports	Daily Temperature (mean), wind speed, relative humidity	Ecological–time series analysis (GAM)	Mean temperature negatively correlated with COVID-19 cases, with 1 °C increase, cases decreased by 13.53% (95% CI: 1.53–4.63) in the range of −2.42 °C–33.37 °C. Average wind speed positively correlated, 1% increase in average wind speed (m/s) associated with 11.21% (95%CI: 0.51–1.19) increase in confirmed cases. Relative humidity (%) not significant.	P values and 95% CI reported. GAM for lag/incubation period effects and non-linear relationship	Confounders not identified. Regional variations in testing and reporting.
Alkhowailed et al. 2020 [44]	Inform Med Unlocked	Saudi Arabia (urban)	5 March 2020–7 June 2020 (95 days)	Daily new cases from Saudi Arabia Ministry of Health dashboard	Temperature, Dew point, Relative Humidity, Wind speed, atmospheric pressure	Ecological study–Spearman’s correlation	Temperature and humidity (weak) negative correlation with new daily cases. Wind speed also negatively correlated with new daily cases.	P values and 95% CI reported Longer time-period	Incubation period/lag not accounted for. Testing limitations. Population density may confound results
Bashir et al. 2020 [33]	Sci Total Environ	New York City, USA (urban/metropolitan)	1 March 2020–12 April 2020 (43 days)	Total confirmed cases and daily new cases from the New York City health department COVID-19 data archive	Temperature, Relative Humidity, Wind speed, Precipitation/rainfall	Ecological study–Spearman’s correlation	Average temperature, minimum temperature and air quality significant correlated with new cases and mortality. Temperature range −3.37 °C–25 °C	A large number of climate variables analyzed in the model	Air quality (confounder) Potentially limited testing and reporting. Short time-period (~one month of data) No accounting for onset/incubation period lag
Briz-Redón & Serrano-Aroca, 2020 [48]	Sci Total Environ	Spain (urban)	25 February 2020–28 March 2020 (33 days)	Daily cumulative cases from online resource	Temperature	Ecological study–spatio-temporal	No consistent evidence for association between temperature and cumulative cases in the temperature range −3.19–29.26	Onset/incubation period lag included in model. Population density, age distribution, travelers and companies by province.	Short study period–2 weeks during lockdown period. Early in pandemic–limited testing and reporting ability Only assessed temperature
Bukhari et al. 2020 [29]	Int J Environ Res Public Health	Global (urban)	20 January 2020–1 May 2020 (103 days)	Confirmed cases from John Hopkins University Coronavirus Resource Centre repository	Temperature, relative humidity, absolute humidity, wind speed.	Ecological study—correlation	For the time-period, the majority of new cases were reported in regions with a mean temperature between 0 °C–17 °C and absolute humidity between 1–9 g/m³. In regions >17 °C and AH >9 g/m³ reported cases much lower for the same time-period.	Large global data set–data at country or state level	Testing and under-reporting of cases, population density, community structures, socioeconomic factors Lag/incubation period not accounted for
Chien & Chen, 2020 [35]	Stoch Environ Res Risk Assess	USA (urban–rural)	16 March–22 April 2020 (38 days)	Confirmed cases from Johns Hopkins Coronavirus Resource Centre	Temperature, Relative Humidity, Precipitation/Rainfall	Ecological study—GAM	Temperature and precipitation significant negative correlation with COVID-19 cases. RR% −0.21 (95%CI: −0.26, −0.15), in further modelling a threshold of 15.3 °C was identified, where RR% switched from negative to positive above this threshold and peaking at 20.25 °C and decreasing towards 29.2 °C. Relative humidity significant positive correlation with COVID-19 cases	GAM–modelling for spatial and temporal factors including lag/incubation period. Included confounders in model	Confounding variables–county-specific population, age, gender, racial composition and poverty level in the model. Limited testing and reporting. Short study period
Goswami et al. 2020 [45]	Diabetes Metab Syndr	India (urban–rural)	1 April 2020–10 May 2020 (40 days)	Confirmed cases from official reports of the Ministry of Health and Family Welfare of India	Temperature, relative humidity	Ecological study—GAM	Statistically significant relationship between cases as a result of interaction between AT and ARH on COVID-19 incidence. Not consistent across regions of India.	Used 3 day moving average to account for lag.	Large geographical area and varying climate range, early strict lockdowns Limited testing
Huang et al. 2020 [30]	Sci Total Environ	Global (urban)	21 January 2020–6 May 2020 (107 days)	Johns Hopkins University	Temperature, Relative humidity, Absolute humidity	Ecological–time series analysis	Significant association between temperature and AH—60% of cases occurred in the temperature range 5–15 °C, cases peaked at 11.54 °C. 73.8% of confirmed cases in regions with AH of 3–10 g/m³.	Large data set with cases from 185 countries/regions. Data from a longer time-period (~3 months)	Correlation between temperature and cases as a distribution rather than assessing association. Doesn’t account for lag or imported cases. Control measures and public health measures
Liu et al. 2020 [36]	Sci Total Environ	China (urban/metropolitan)	20 January 2020–2 March 2020 (43 days)	Daily confirmed cases from Health Commissions per city	Temperature, humidity, diurnal temperature range	Ecological study—GLM	AT, AH and DTR negatively associated with transmission in pooled results. AT increase of 1 °C correlated to decrease in daily case counts RR = 0.80 (95% CI: 0.75–0.85) in the range −20 °C–20 °C. 1% increase in DTR associated with decrease in lag cases RR = 0.90 (95% CI: 0.86–0.95). increase in AH of 1 g/m³ associated with decrease in cases RR = 0.72 (95% CI: 0.59–0.89) and 0.33 (95% CI: 0.21–0.54) based on lag period.	Accounted for lag using 0, 3, 7 and 14-day intervals.	Migration Scale Index (MSI)
Menebo, 2020 [51]	Sci Total Environ	Oslo, Norway (urban/metropolitan)	27 February 2020–2 May 2020 (66 days)	Daily cases from the Norwegian public health institute	Temperature, Precipitation/rainfall, Wind speed	Ecological study–Spearman’s rank correlation	Tmax (*r* = 0.347; *p* = 005) and Tavg (*r* = 0.293 *p* = 0.019) significant positive correlation with COVID-19 cases in the temperature range −0.5 °C–21.9 °C. Precipitation significant negative correlation with COVID-19 cases.	Lag analysis of 5,6,14 days for weather variables and onset	Humidity not included in model Public health measures–lockdowns, sanitization, testing capabilities not included in analysis
Meraj et al. 2020 [46]	Environ Dev Sustain.	India (urban–rural)	9 March 2020–27 May 2020 (80 days)	Cumulative cases from official region web sites for Maharashtra, Kashmir and Rajasthan	Temperature	Ecological study–Pearson’s correlation	Significant positive correlation with COVID-19 cases in Rajasthan (25 °C–45 °C) and Kashmir (10 °C–32 °C). No significant association between temperature and COVID-19 in Maharashtra (29 °C–38 °C).	Comparison between provinces with varying climates and ecologies	Only temperature included for three provinces. Limited testing rates initially (increased over time).
Meyer et al. 2020 [32]	Front public Health	Global (100 countries)(Urban)	29 December 2020–17 March 2020 (80 days)	Daily cases from WHO daily reports	Temperature, Humidity	Population–cohort study GLMM	Statistically significant association with temperature and COVID-19 incidence in temperature range −33.9–34.3 °C). Small effect size.	Lag delay between 3 and 20 days in model. Cases identified as local or imported.	Limitations in COVID-19 testing and reporting early in pandemic.
Paez et al. 2020 [47]	Geogr Anal.	Spain (urban)	13 March 2020–11 April 2020 (30 days)	Daily new cases at provincial level from Centro de Datos COVID-19	Temperature, Humidity, Daily Sunshine	Ecological study–Spatio-temporal	Temperature significant negative correlation with COVID-19 incidence in the range 1 °C–23.2 °C, humidity significant negative correlation when accounting for control variables.	Confounders included in SUR model as controls Spatial and temporal modelling. Lag/incubation period included	Confounding variables–GDP, age, population density, province area. Human behavioral changes as a result of lockdown, hours of sunshine and population density
Pani et al. 2020 [50]	Sci Total Environ	Singapore (urban/metropolitan)	23 January 2020–31 May 2020 (59 days)	Daily cases of new infections and deaths from the Ministry of Health (MOH)	Temperature, Relative humidity, Absolute humidity, Surface pressure, Dew point, Wind speed, Water vapor	Ecological study–Spearman’s rank correlation	Temperature significant positive association with daily and cumulative cases in the range 24 °C–32 °C. TRH significant for transmission, along with AH and WV. WS not significant.	Large range of weather variables included in model	Meteorological data limited to one site. Public health measures and personal hygiene. Testing limitations.
Prata et al. 2020 [41]	Sci Total Environ	Brazil (urban)	27 February 2020–1 April 2020 (35 days)	Daily cumulative cases reported by the Ministry of Health of Brazil	Temperature	Ecological study—GAM	For each 1 °C rise in temperature, daily COVID-19 cases decreased by 4.9% when temperature below 25.8 °C, in the range of 16.8 °C–27.4 °C.	Climate and temperature zones, GAM and GLM analysis	Testing limitations, only temperature included
Qi et al. 2020 [38]	Sci Total Environ	China (urban)	1 December 2019–11 February 2020 (73 days)	Daily confirmed cases from National Health Commission	Temperature, Relative Humidity	Ecological study–time series analysis GAM	Temperature and Humidity have a significant negative correlation with COVID-19 incidence in the range 1.5 °C–11.42 °C.	Data from early outbreak in China prior to travel restrictions. GAM with internet search results for health-seeking behaviors. 14-day onset lag/incubation period	Short study period early in outbreak Confounders not included in model–socioeconomic status, intervention measures, weather is at city, not province level
Shi et al. 2020 [39]	Sci Total Environ	China (urban)	20 January 2020–29 February 2020 (41 days)	Confirmed cases from China National health Commission (CNHC)	Temperature	Ecological–spatio-temporalSEIR model	Temperature had a significant effect on COVID-19 incidence in the range −22 °C–26 °C. Increasing temperature associated with decreased infection rate. RR = 0.96 (95%CI: 0.93–0.99)	Dynamic transmission model with lag/incubation period delay	Only temperature included. Limited study period early in outbreak. Limited testing and reporting, change in diagnostic criteria–healthy patients excluded.
To et al. 2020 [34]	Sci Total Environ	Canada (urban–rural)	25 January 2020–18 May 2020 (115 days)	Case data for Alberta, British Columbia, Ontario and Quebec	Temperature, Precipitation/rainfall, Wind speed	Ecological study–linear regression model	No significant association for Temperature and COVID-19 incidence when adjusted for wind speed, precipitation and province in the temperature range −6.83 °C–7.94 °C	A large geographical area with variations in temperature across the country, 2-week lag delay for climate variables	Model adjusted for wind speed, precipitation and province to evaluate temperature Linear regression model. Local public health policies, testing rates and urbanization varies across regions
Tosepu et al. 2020 [49]	Sci Total Environ	Indonesia (urban/metropolitan)	January 2020–29 March 2020 (~29 days)	Daily COVID-19 cases from the Ministry of Health of Republic of Indonesia	Temperature (min, max, avg), Humidity, Precipitation/Rainfall	Ecological study–Spearman’s rank correlation	Average temperature correlated with COVID-19 cases (26.1 °C–28.6 °C). Tmin, Tmax, humidity and rainfall not significantly correlated.	Large range of weather variables	High mobility and population density in Jakarta Small data set/limited testing, no accounting for lag/incubation period
Ward et al. 2020 [42]	Transbound Emerg Dis	Australia (urban)	12 February 2020–30 March 2020 (35 days)	NSW Government Case Reports	Temperature, Relative humidity, Precipitation/Rainfall	Ecological study–time series analysis GAM	Negative significant relationship between relative humidity and COVID-19 cases (*p* = 0.0304) where a 1% decrease in morning humidity associated with up to 6.11% increase in cases. Temperature (18.4 °C–25.5 °C) and rainfall were not significant in time series analysis.	Good quality data set available, high testing rate and reporting. Accounted for 14-day lag/incubation period	Limited case numbers available, mostly imported cases rather than local transmission
Wu et al. 2020 [31]	Sci Total Environ	Global (166 countries excluding China) (urban)	Up to 27 March 2020 (~75 days)	Daily cases and deaths from WHO COVID-19 daily situation reports	Temperature, Dew point, Wind speed, Relative humidity	Ecological study–time series analysis GAM	Temperature and RH negatively correlated to daily cases. 1 °C increase in temperature associated with 3.08% (95% CI: 1.53–4.63%) reduction in cases in the range of −5.28 °C–34.3 °C. 1% increase in RH associated with 0.85% (95% CI: 0.51–1.19%) reduction in new daily cases.	Global data set with daily new cases and deaths. Included confounders in modelling–economic level, additional health conditions, age and density. Daily lag/incubation period included in model	Confounders—Wind speed, median age, Global Health Security Index, Human Development Index, population density
Xie & Zhu, 2020 [37]	Sci Total Environ	China (urban)	23 January 2020–29 February 2020 (38 days)	Daily cases from official websites of each city heath commission.	Temperature, Relative Humidity, Air Pressure, Wind speed	Ecological study– exposure response GAM	Exposure response positive linear when temperature below 3 °C and flat above 3 °C. Each 1 °C rise in temperature was associated with 4.861% (95%CI:3.209–6.513) increase in COVID-19 cases average daily temperature range −33.8 °C–26.9 °C	Large number of variables included in model. Lag/incubation period from 0–7, 0–14, 0–21 days	Case data from early in outbreak, limited testing and diagnostic capabilities. Short study period
Zhu et al. 2020 [40]	Sci Total Environ	South America (urban/metropolitan)	23 February 2020–6 May 2020 (74 days)	Data from National health departments or secondary websites	Temperature. Wind speed, Relative Humidity	Ecological study–Spearman’s rank correlation	Large variation in correlation by region. Daily average temperature and absolute humidity were most strongly correlated with COVID-19 cases averaged across regions. (Temperature range 3.9 °C–35 °C) Wind speed not significantly correlated with daily cases.	Accounted for lag using daily incubated cases (median ~4 days prior to positive test)	Demographic, geographical and socioeconomic factors, healthcare infrastructure, governmental and social policies, testing and reporting.

## Data Availability

No new data were created or analyzed in this study. Data sharing is not applicable to this article.

## Supplementary Materials

The following are available online at https://www.mdpi.com/1660-4601/18/2/396/s1, Table S1: Quality Assessment.
