# Supplementary material for: Weather Variability and COVID-19 Transmission: A Review of Recent Research
Source: Int J Environ Res Public Health. 2021 Jan 6;18(2):396. doi: 10.3390/ijerph18020396 (PMC7825623; doi:10.3390/ijerph18020396)
Supplement: Supplementary file 1 [file ijerph-18-00396-s001.pdf]

Supplementary Materials

Table S1: Quality Assessment

Quality Assessment		
**Criteria**	**Question**	**Score**
**Source of information** (1-5**)**	Published in a peer-reviewed journal? Yes/No	1/0
	Are all investigators and institutional affiliations identified? Yes/No	1/0
	How long was information collected for?	
	More than 1 month	3
	1 week to 1 month	2
	1 week	1
**Study Design** (0-11**)**	Study design described clearly? Yes/No	1/0
	Is the design appropriate to the study question? Yes/No	1/0
	Are weather variables (Temperature, Humidity, Wind speed, Rainfall) clearly defined, including units of measurement? Yes/no	1/0
	Are non-meteorological variables being assessed? (population density, visitors, population movement) Yes =0/No = 1	1/0
	Are there appropriate statistical packages for data analysis? Yes/No	1/0
	Are spatial or temporal methods (mapping or modelling) used? Yes/No	1/0
	Controlled for potential confounders (testing lag, symptom onset, imported vs community transmission etc.)? Yes/No	

		1/0
	Is the outcome (COVID-19) clearly defined using ICD9 or ICD10?	
	Yes/No	1/0
	How is COVID-19 data reported?	
	Daily new cases	3
	Cumulative cases	2
	Rt value or mortality	1
**Study Results** (0-3)	Is the main question/hypothesis of the study answered properly?	
	Yes/No	1/0
	Are OR/RR, 95% CI, p values represented?	
	Yes/No	2/0
**Study Discussion** (0-3)	Limitations/innovation of the study discussed?	
	Yes/No	1/0
	Comparison with previous work?	
	Yes/No	1/0
	Discuss plausibility?	
	Yes/No	1/0
**Total Score**		**Min = 1 Max = 22**
